# Epigenetic alterations to Polycomb targets precede malignant transition in a mouse model of breast cancer

**DOI:** 10.1038/s41598-018-24005-x

**Published:** 2018-04-03

**Authors:** Ying Cai, Jhih-Rong Lin, Quanwei Zhang, Kelly O’Brien, Cristina Montagna, Zhengdong D. Zhang

**Affiliations:** 1 Department of Genetics, Albert Einstein College of Medicine, Bronx, New York USA; 2 Department of Pathology, Albert Einstein College of Medicine, Bronx, New York USA

## Abstract

Malignant breast cancer remains a major health threat to women of all ages worldwide and epigenetic variations on DNA methylation have been widely reported in cancers of different types. We profiled DNA methylation with ERRBS (Enhanced Reduced Representation Bisulfite Sequencing) across four main stages of tumor progression in the MMTV-PyMT mouse model (hyperplasia, adenoma/mammary intraepithelial neoplasia, early carcinoma and late carcinoma), during which malignant transition occurs. We identified a large number of differentially methylated cytosines (DMCs) in tumors relative to age-matched normal mammary glands from FVB mice. Despite similarities, the methylation differences of the premalignant stages were distinct from the malignant ones. Many differentially methylated loci were preserved from the first to the last stage throughout tumor progression. Genes affected by methylation gains were enriched in Polycomb repressive complex 2 (PRC2) targets, which may present biomarkers for early diagnosis and targets for treatment.

## Introduction

DNA methylation is one of the epigenetic marks that regulate gene expression^1^. Alterations in DNA methylation are commonly observed in various cancer types^2–5^ and a well-known signature of the cancer genome is global hypomethylation accompanied by promoter hypermethylation of some tumor suppressor genes^6–11^. Some genes in tumors showed distinct hypomethylation compared to normal cells^10^ and cancer progenitor cells with such DNA methylation alterations may help to predict cancer risks^11^. Global hypomethylation has been proposed as a mechanism driving chromosomal instability and elevated mutation rates^12,13^ and hypermethylation caused gene suppression, such as *HAND2*, is suggested to contribute to cancer development^14^; hence DNA methylation abnormalities have been considered to play a causal role in tumorigenesis^15^.

Despite extensive profiling of gene expression and DNA methylation changes in breast cancer, not much is known about the dynamics of DNA methylation during cancer progression. It is almost impossible to monitor tumor progression by following the same patients, a task that can be easily fulfilled by using mouse models with almost identical genetic backgrounds. A better understanding of DNA methylation dynamics in breast carcinogenesis is not only vital to explain transcriptional deregulation of gene expression during tumor progression, but also adds to the understanding of tumor class and subtypes^16,17^ and prognosis^18^. Abnormal DNA methylation has been reported in breast carcinoma with subtype specific patterns^19^. In particular, the more aggressive luminal B subtype shows higher DNA methylation when compared with other subtypes and normal tissues^19,20^; and reduced gene expression correspondingly^20^.

Human breast cancer begins with premalignant atypical ductal hyperplasia (ADH), moves to the ductal carcinoma *in situ* (DCIS), and progresses to invasive ductal carcinoma (IDC)^21,22^. Despite extensive evidences suggesting that ADH and DCIS are precursors of IDC, DNA methylation biomarkers identified at the early stages of breast cancer are limited. This represents a significant gap of knowledge in the field since such biomarkers may explain the biological basis of tumor progression and helps to predict outcomes. A recent study analyzing DCIS and IBC (invasive breast carcinoma) samples identified only 18 CpG loci associated with survival and prognosis of breast cancer patients^18^. Another study using FEA (flat epithelial atypia), ADH, DCIS, and IDC found increased number of genes with elevated promoter methylation during the progression^23^. Although it has been discovered that normal tissue adjacent to breast cancer already exhibited numerous DNA methylation alterations^24^, how those alterations change during progression is not well defined yet. Thus, it is apparent that early DNA methylation changes are relevant to breast carcinogenesis but their characterization is lacking. It is critical to comprehensively examine DNA methylation during the early stages of breast cancer progression.

The MMTV-PyMT transgenic mouse is an ideal model to study tumor progression because primary tumors evolve from pre-malignancy to an invasive malignant tumor through four stereotypical stages of progression (hyperplasia, adenoma/mammary intraepithelial neoplasia (MIN), early carcinoma and late carcinoma). Breast tumors in the PyMT mouse share both morphological and transcriptional similarities with human breast tumors^25^, and generally cluster with the human luminal B subtype^26,27^. Previous cytogenetic profiling also showed similarity of breast tumor from PyMT mice to human breast carcinoma on the level of chromosomal structural and numerical abnormalities^28,29^.

For the first time, we report the dynamics of DNA methylation deregulation from early hyperplasia to late carcinoma across the four main stages of breast tumor progression. We performed ERRBS (Enhanced Reduced Representation Bisulfite Sequencing), which quantitatively measures DNA methylation at the base-pair resolution and increases coverage on CpG sites relative to RRBS^3^. We observed, as expected, a global hypomethylation and a shift towards hypermethylation as the tumors progressed. We found genes with promoter hypermethylation enriched in Polycomb repressive complex 2 (PRC2) targets throughout tumor progression. These shed lights on the mechanisms of abnormal DNA methylation, using the PyMT mouse model of breast cancer progression.

## Results

### DNA methylation profiles of the PyMT mouse model unveil DNA methylation changes precede malignant transition

**Figure 1:**
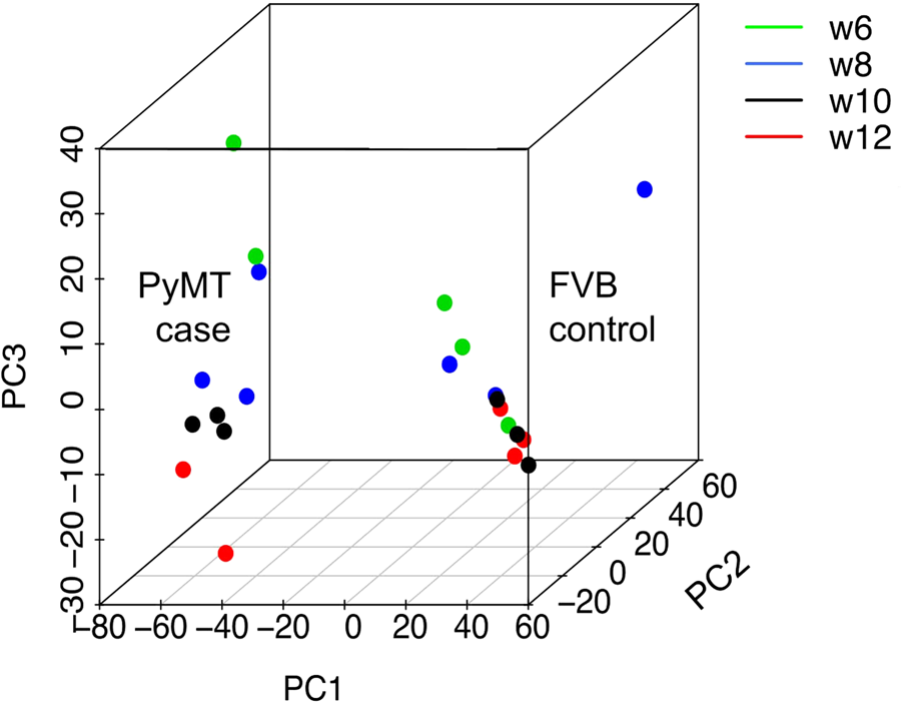
Principal component analysis of DNA methylation profiles of both PyMT and FVB control samples. Samples were taken at four time points: weeks 6, 8, 10, and 12 – shown as w6, w8, w10, and w12 – correspond to hyperplasia, adenoma/MIN, early carcinoma and late carcinoma in PyMT mice.

We first profiled DNA methylation alterations in hyperplasia, adenoma/MIN, early carcinoma and late carcinoma samples from the PyMT mice and normal tissues from FVB controls with principal component analysis (PCA). After removing two outlier samples, the PyMT samples clearly separated from the FVB controls, as shown in Fig. 1. In addition, the tumor samples separate across their temporal stages, whereas the controls remain within one cluster. This pattern is consistent with the PCA results from gene expression profiles of this mouse model as we reported previously^30^. Only biological covariates but not technical ones contribute to PCs (Supplementary Fig. S1), suggesting the variance observed here was not due to batch effect but was of biological meanings.

**Figure 2:**
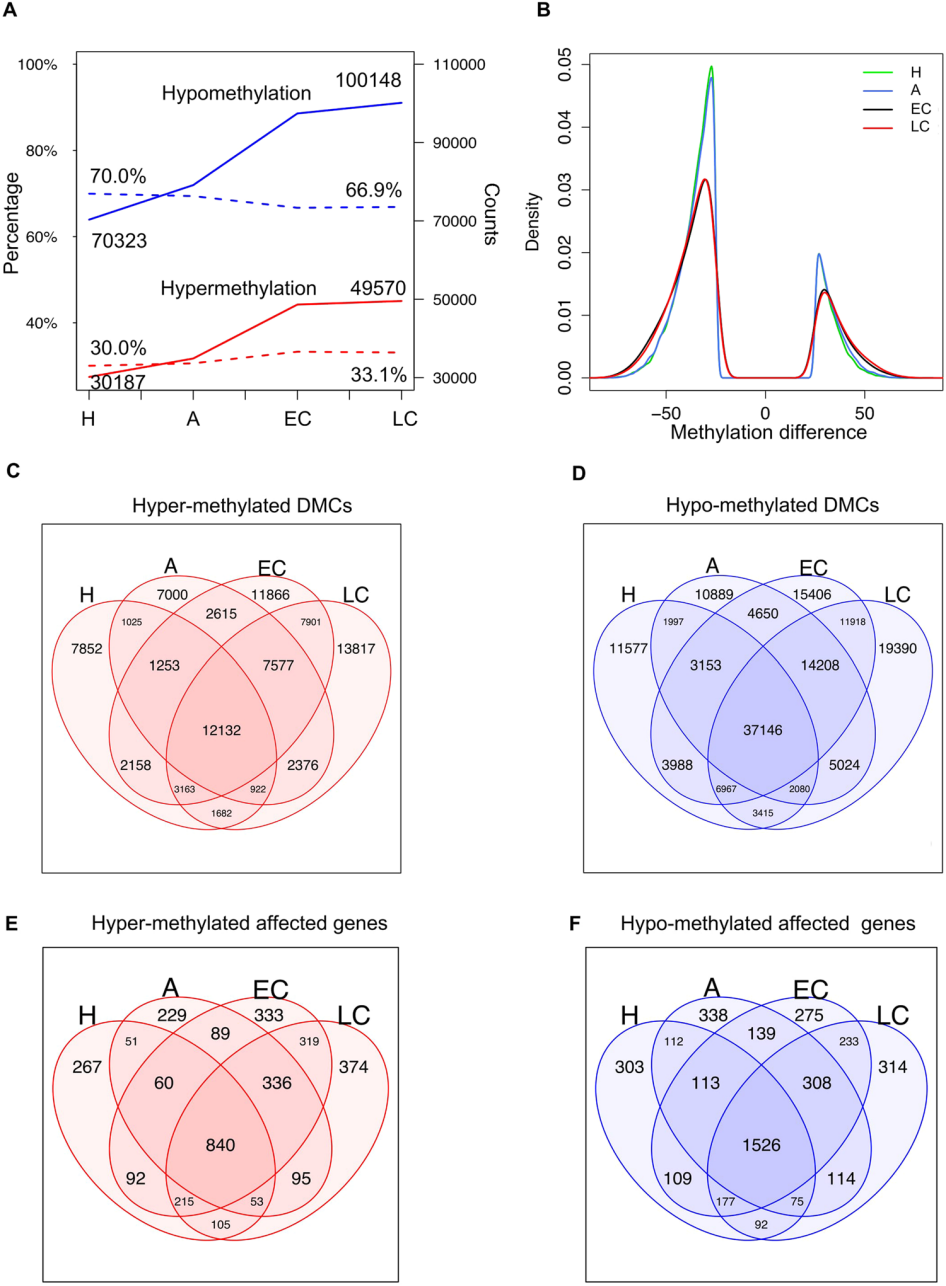
DMCs at different stages of tumor progression. (**A**) Numbers of DMCs. Counts and percentages are connected by solid and dashed lines, hypermethylation and hypomethylation are represented by red and blue lines; w6, w8, w10, w12 as week 6, week 8, week 10 and 12, corresponding to hyperplasia, adenoma/MIN, early carcinoma and late carcinoma. (**B**) DMCs (methylation difference ≥25% and *q*-value < 0.01) methylation difference distribution at four time stages. (**C**,**D**) Intersections of DMCs. (**E**,**F**) Intersections of genes whose promoter contain DMCs. H = hyperplasia, A = adenoma/MIN, EC = early carcinoma, LC = late carcinoma.

As tumors progressed from hyperplasia to late carcinoma, the number of DMCs (methylation difference ≥10% and *q*-value < 0.01, Methods) increased for both hyper- and hypo-methylation, but hypomethylated loci were consistently more frequent (Table 1 and Fig. 2A). Interestingly, we observed a shift toward hypermethylation by roughly 3% (30.0% in hyperplasia to 33.1% in late carcinoma, DMC-hypermethylation percentage + DMC-hypomethylation percentage = 1) (Fig. 2A). The late stages (early carcinoma and late carcinoma) were similar on methylation patterns yet distinct from early stages (Fig. 2B).

**Table 1:** Total DMCs.

Stage	Hypomethylation	Hypermethylation	Total
Hyperplasia	70,323	30,187	100,510
Adenoma/MIN	79,147	34,900	114,047
Early carcinoma	97,436	48,665	146,101
Late carcinoma	100,148	49,570	149,718

To investigate impact of DNA methylation on gene expression and the possible gene affected, we mapped DMCs to the *cis*-regulatory elements of promoters (±2 kb to RefSeq TSS) and enhancers (if the DMCs overlapped with predicted enhancer regions, Methods). Consistent with the high preservation of DMCs between neighboring stages and among all stages (Fig. 2C,D), we found that gene promoters overlapping with these DMCs were largely the same (Fig. 2E,F). These observations provide evidence that DNA methylation changes at late stages can be traced back to early lesions. Interestingly, using curated gene lists from MSigDB (Molecular Signatures Database) and GSEA (gene set enrichment analysis)^31^, we found an enrichment of PRC2 targets (FDR < 0.05) among the 374 genes with promoter hyper-DMCs unique to late carcinoma stage (*Barhl2, Cacna1e, Cntfr, Col4a6, Dkk2, Dok6, Elmod1, Fezf2, Fli1, Fzd10, Gabra2, Gdf7, Gria2, Gsc, Hlx, Hoxb7, Hoxb8, Hoxc12, Hoxd13, Hrk, Isl1, Lhx8, Neurod1, Nkx2-1, Nkx2-2, Nkx3-2, Nkx6-2, Otop3, Pdx1, Ripk3, Sctr, Tal1* and *Wt1*) and among the 840 genes with promoter hyper-DMCs common to all four stages (including *Alx3, Cited1, Comp, Coro6, Crlf1, Csmd1, Cyp26c1, Dgki, Dll4, Duoxa1, Eomes, Esam, Gdnf, Hes7, Hhex, Hhip, Hmx2, Hoxc5, Hoxc6, Hsf4, Il1rapl2, Lrfn5, Ltk, Mab21l1, Nfix, Nkx2-8, Nrg1, Otx1, Pax7, Podn, Ptgdr, Rasl10a, Sidt1, Slc10a4, Slc1a2, Sox7, Tbr1, Tcea3, Tmem59l, Tmem88, Tp73, Ucn* and *Ush1g*). The former are potential biomarkers that distinguish late carcinoma from early stages and the latter can be potential biomarkers for early cancer detection. The distributions of CpGs captured and DMCs mapped to genomic compartments were similar across different stages. About 10% of all CpGs captured and about 26% (26% at hyperplasia, 23% at late carcinoma) of DMCs were mapped to putative enhancers (Supplementary Fig. S2A), suggesting DMCs were enriched in enhancers (permutation test *p*-value < 0.05, Supplementary Fig. S2B, Methods). About 14% of DMCs and 28% of hypermethylated DMCs were mapped to promoters (Supplementary Fig. S2C), suggesting hypermethylation is biased toward promoters (permutation test *p*-value < 0.05, Supplementary Fig. S2D). Not many hypomethylated DMCs (and DMCs overall) were detected in the promoter regions, possibly due to their low methylation in general.

**Figure 3:**
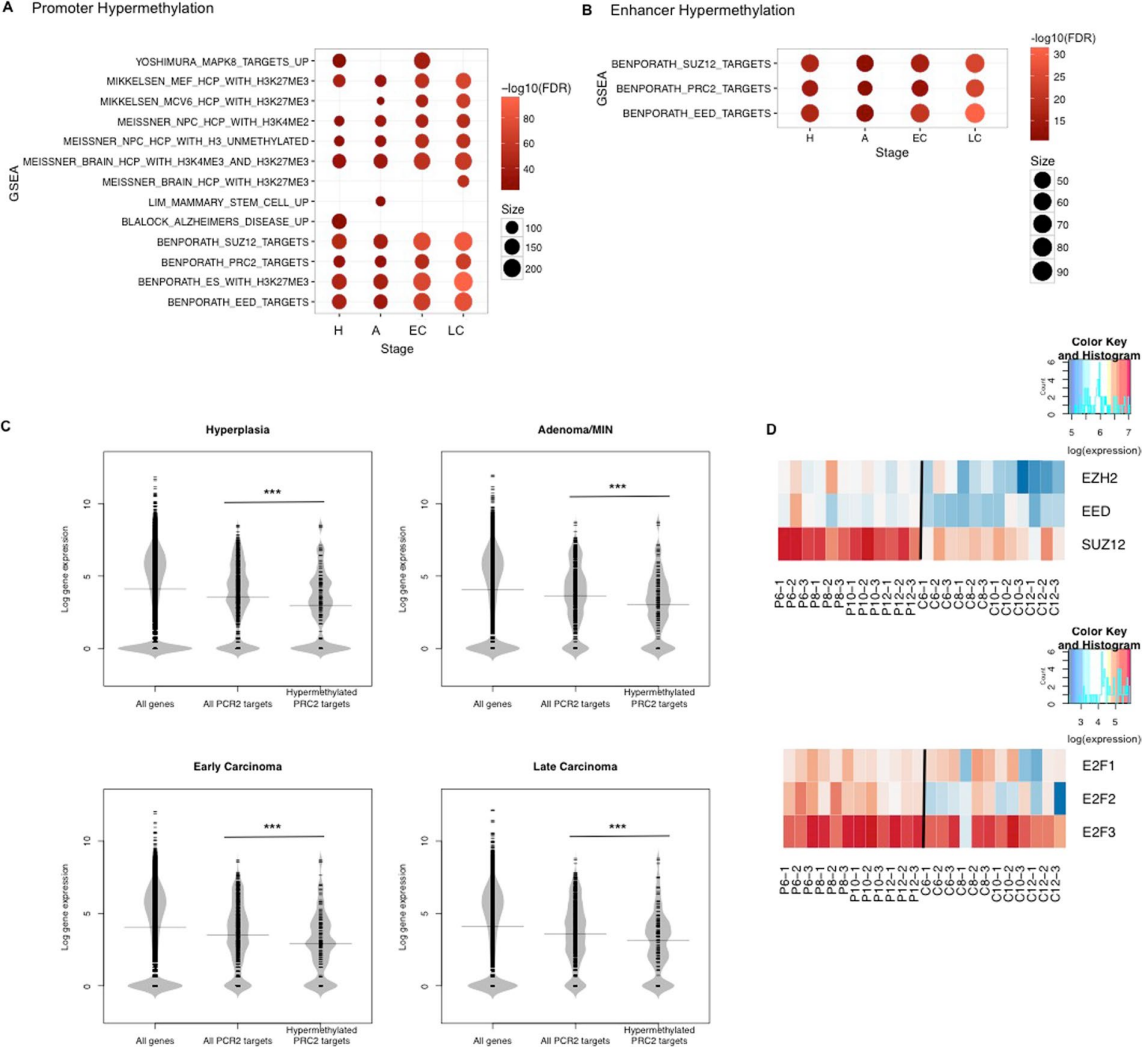
PRC2 targets enrichment and expression. (**A**) Gene set enrichment analysis with GSEA on genes with promoter hypermethylation. The top 10 terms with most significant FDR at each stage were plotted. H = hyperplasia, A = adenoma/MIN, EC = early carcinoma, LC = late carcinoma. Color represents –log_10_(FDR), dot size represents the number of genes overlapping each term. (**B**) Gene set enrichment analysis with GSEA on hypermethylated enhancer target genes. (**C**) Log transformed gene expression of PRC2 targets with promoter hypermethylation (at week 12) compared with all PRC2 targets and all genes. (***Wilcoxon-test *p*-value < 0.05) (**D**) Log transformed gene expression of EZH2, EED and SUZ12. (**E**) Log transformed gene expression of E2Fs. P = PyMT, C = controls, 1–3 represent 3 biological replicates.

We then analyzed the pathway enrichment among genes linked to promoters and enhancers with hypermethylated DMCs (Fig. 3A,B), assuming nearest genes within 10 kb of an enhancer as its targets. Genes with hypermethylated DMCs in promoters were enriched in targets of PRC2, EED, and SUZ12 (Fig. 3A, FDR < 0.05). Putative targets of hypermethylated enhancers were also enriched in PRC2 targets (Fig. 3B, FDR < 0.05). We further observed a significant reduced expression of such PRC2 target genes with promoter hyper-DMCs comparing to all PRC2 targets in all four stages (Fig. 3C, Wilcoxon-test *p*-value < 0.05). In addition, three core components of PRC2 (EZH2, EED and SUZ12) all exhibited higher mRNA expression in PyMT samples than in controls (Fig. 3D). Two core components of PRC2 (EHZ2 and EED) are E2F targets and we observed higher expression of E2Fs in the PyMT mice as well (Fig. 3E).

To further study how gene expression is affected by DNA methylation in each cancer developmental stage, we analyzed together genes with promoter DMCs identified in this study and differentially expressed genes (FDR < 0.05) that we identified earlier in our study of mRNA expression of this PyMT mouse model^30^. Comparing PyMT samples from the four stages with age-matched normal tissues from controls, we identified 101, 109, 159, and 198 genes, respectively, with both hypomethylated DMCs in promoter and increased gene expression and 131, 152, 216 and 249 genes with both hypermethylated DMCs in promoter and reduced gene expression. Down-regulated genes with promoter hypermethylation were enriched with genes involved in epithelial mesenchymal transition (EMT) in week 10 (FDR < 0.05, *Tagln, Sgcb, Col4a2, Wipf1, Tpm2, Igfbp3, Mest, Col1a1, Tpm1, Mmp14, Fstl1, Fap, Plod1*) and week 12 (FDR < 0.05, *Htra1, Mest, Slit3, Timp3, Tagln, Col4a1, Sgcb, Dpysl3, Tpm2, Col1a1, Wnt5a, Col1a2, Emp3, Fap, Nid2, Fstl1, Tpm1*). Text mining of PubMed literature with an R package RISmed revealed that most of them are with unclear roles in breast cancer except for *Igfbp3, Mmp14, Fap, Timp3* and *Wnt5a*.

### Time-course analysis of DMRs reveals increasing methylation differences as tumor progress

**Figure 4:**
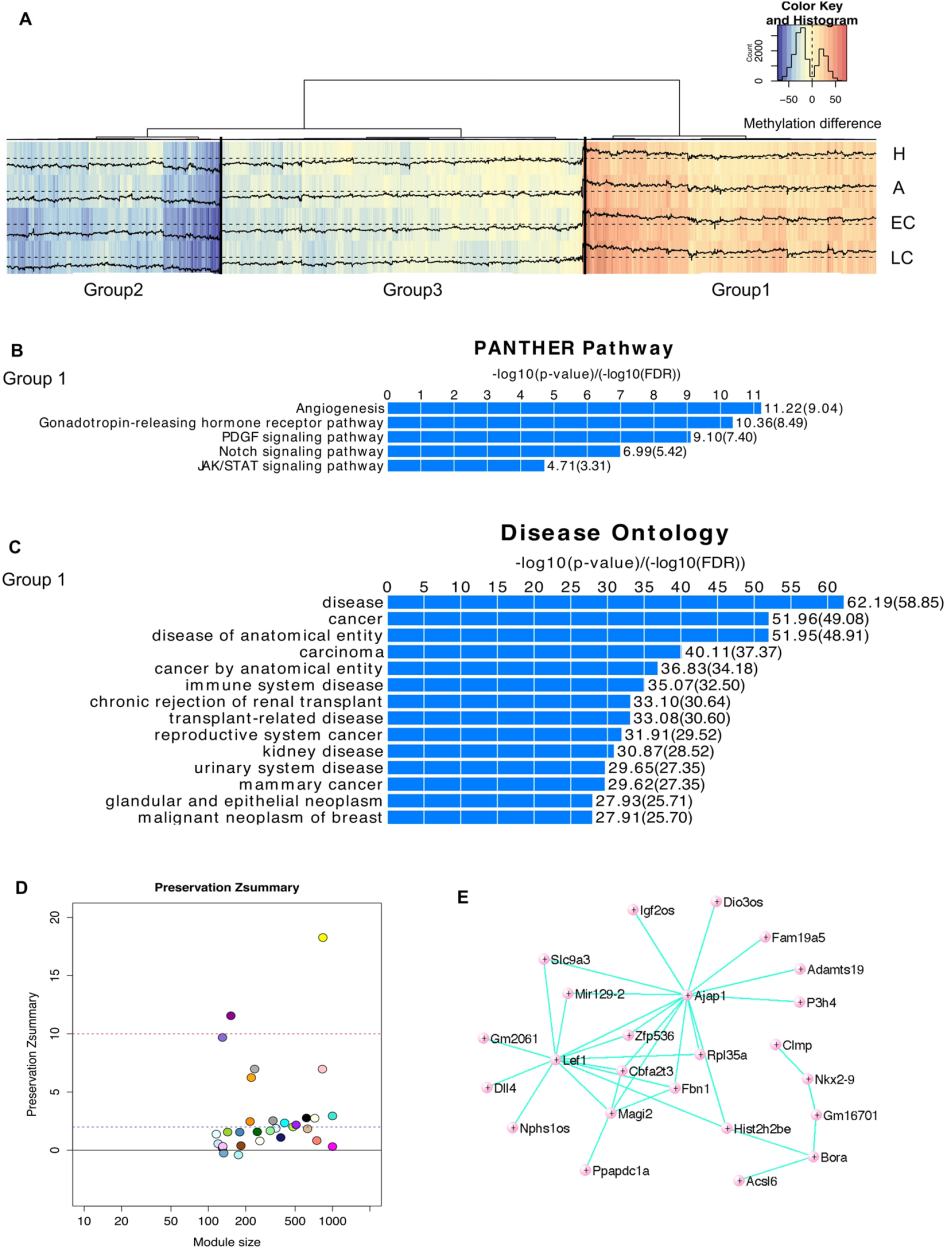
Clusters and enrichment of DMRs. (**A**) Hierarchical clustering of all DMRs detected during cancer progression. Color represents mean methylation differences between PyMT samples and controls. Dashed black lines represent 0 values, solid lines are mean values of methylation difference. (**B**) PANTHER pathway enrichment of Group 1. (**C**) Disease ontology enrichment of Group 1. H = hyperplasia, A = adenoma/MIN, EC = early carcinoma, LC = late carcinoma. (**D**) Preservation z-summary scores for all moduels detected. (**E**) Some hub genes of the pink module, visualized with VisANT^88^.

Since a dense region of differentially methylated loci may also have a regulatory impact on TF binding and gene expression, we identified differentially methylated regions (DMRs) – i.e., dense regions of DMCs – using an R package eDMR (with default settings, DMR mean methylation difference ≥20%)^32^. DMRs from various stages have a similar length distribution (median = ~185 bp) and the numbers of DMRs increased as tumor progressed (Table 2). To study chronological changes of DNA methylation, we analyzed the union of DMRs identified in the four stages (as long as a region was identified as DMR in one stage, we included that region). Unsupervised hierarchical clustering on the union of DMRs revealed three groups (Fig. 4A): groups 1 (1,447) with hypermethylation, group 2 (1,050) with hypomethylation and group 3 (1,790) without significant methylation differences (methylation difference <20%) at the premalignant stages but with a moderate level of hypomethylation at the malignant stages. Then we applied GREAT (Genomic Regions Enrichment of Annotations Tool)^33^ to associate DMRs with genes and test for enrichment. Because hypermethylation is often associated with *cis*-regulatory regions, we are more interested in Group 1. Angiogenesis was one of the significantly enriched terms in Group 1 (Fig. 4B).

**Table 2:** Total DMRs.

Stage	Hypomethylation	Hypermethylation	Total
Hyperplasia	1,447	471	1,918
Adenoma/MIN	1,410	594	2,004
Early carcinoma	2,054	1,004	3,058
Late carcinoma	2,221	1,156	3,377

Genes in Group 1 were enriched in disease ontology terms related to cancer, carcinoma, and neoplasm of breast (Fig. 4C). This suggests the DMRs identified here are of biological significance in breast cancer. We also confirmed the enrichment of PRC2 targets (FDR < 0.05) in this group. In Group 1, we found 30 regions connected to 24 genes in PANTHER angiogenesis pathway (*Fzd1, Angpt2, Wnt5a, Vegfa, Dok3, Grap, Fzd5, Pik3r2, Arhgap8, Wnt7b, Shc1, Notch2, Grb7, Fzd2, Rhoc, Axin2, Pxn, Grb2, Plcg2, Dll4, Pik3c2b, Ephb2, Hras1*, and *Prkcz*), including some *Fzd* genes. We found a hypermethylated DMR in the gene body of *Fzd5* (TSS + 2,655 bp) and overexpression of *Fzd5* in PyMT mice. *Fzd* genes encode Frizzled class receptors, which initiate Wnt signaling cascade when activated. The COSMIC (Catalogue of Somatic Mutations in Cancer)^34^ database reported overexpression of *Fzd5* in 475 TCGA cancer samples. Previous studies also observed hypermethylation in the gene bodies of *Fzd1, Fzd2, Fzd7*, and *Fzd10* in pancreatic adenocarcinoma^35^.

**Figure 5:**
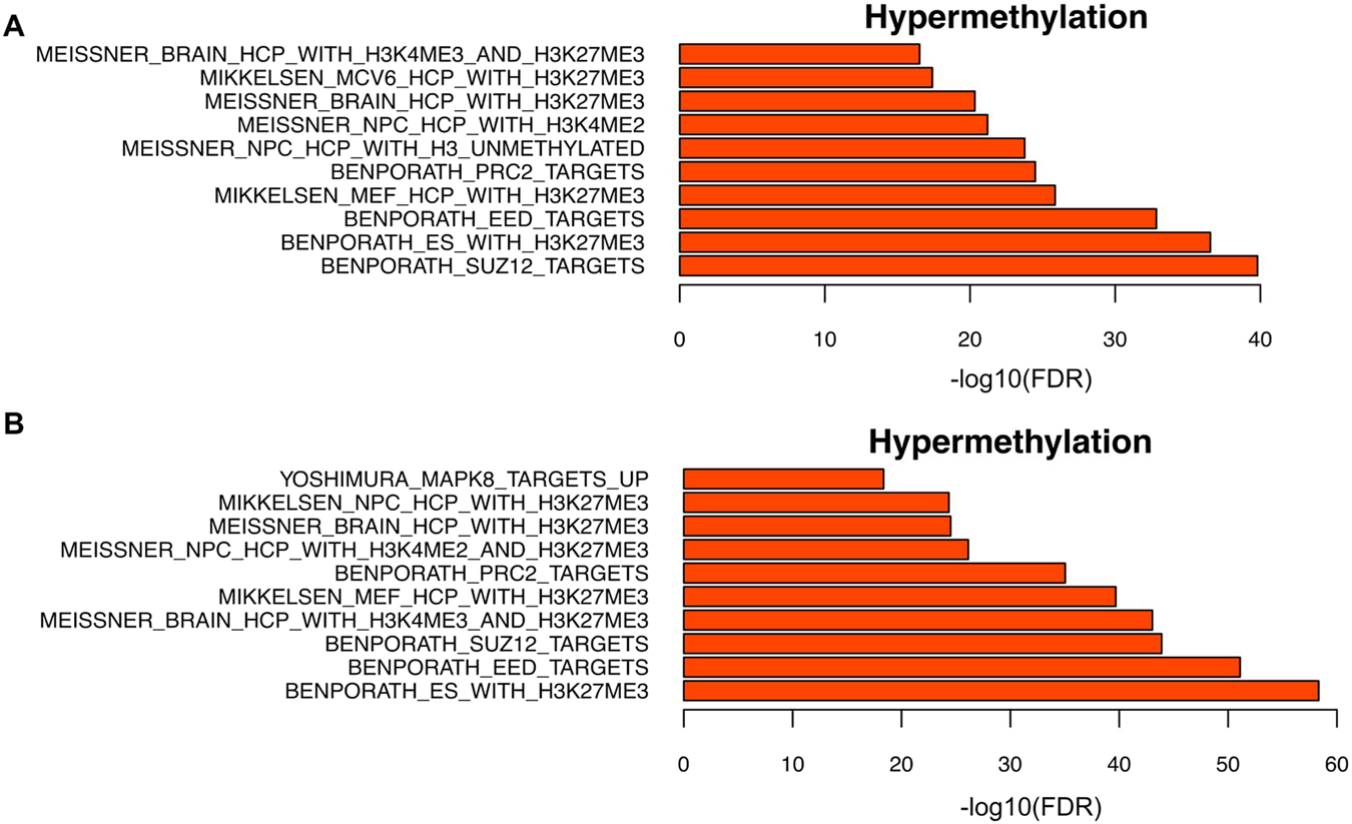
GSEA gene set enrichment. (**A**) Genes with promoter hypermethylation during malignant transition enriched in PRC2 targets. (**B**) Genes with promoter hypermethylation (DMR methylation difference ≥40% and *q*-value < 0.05) in luminal B TCGA samples enriched in PRC2 targets.

In addition, our DNA methylation profiles showed a clear transition from the premalignant to the malignant invasive stages (Fig. 2A,B). To further investigate DNA methylation changes in the malignant transition, we compared all premalignant PyMT samples (weeks 6 and 8) with all malignant samples (weeks 10 and 12) and mapped DMCs (methylation difference ≥10% and *q*-value < 0.01) identified to gene promoters. To minimize effects due to mammary gland development, we filtered out genes that were also identified by a similar comparison using control samples. We found 496 and 943 genes with promoter hypomethylation and hypermethylation, respectively, at the malignant stages compared with early lesions. Those 943 genes were enriched in PRC2 targets (Fig. 5A). The results are consistent with DMC and DMR analysis that genes with increased methylation during cancer progression were enriched in PRC2 targets.

### Co-methylation network analysis

In addition to the differential methylation analyses, we built and explored the co-methylation gene network to investigate gene functions at a higher systems level. We discovered 31 modules, including a module that was highly preserved between PyMT samples and controls as measured by the z-summary score (colored in pink in Fig. 4D) and also showed increased methylation (Wilcoxon test, adjusted *p*-value < 0.05) in PyMT samples. After mapping DMCs in this module to gene promoters, we discovered an enrichment of SUZ12, EED and PRC2 targets (FDR < 0.05) among genes in this module. Moreover, we identified genes – e.g., *Ajap1* and its network neighbors – with strong co-methylation connections in this module (Fig. 4E). Interestingly, the putative tumor suppressor AJAP1 has been suggested to be epigenetically silenced by DNA methylation in many glioblastoma^36^ and its reduced expression might be associated with patients’ better survival outcome^37^.

### TCGA breast invasive carcinoma samples exhibit DNA methylation dysregulation in PRC2 targets

To translate our findings from the PyMT mice to human, we studied TCGA breast carcinoma data of gene expression (RNA-sequencing) and DNA methylation (HumanMethylation450 BeadChip). After subtyping all human breast cancer cases into molecular types using the AIMS method^38^, we end up with 269 luminal A, 189 luminal B, 111 HER2-enriched, and 142 basal-like samples. We used the bump-hunting method implemented in the R package minfi to identify DMRs (methylation difference ≥25% and FDR < 0.01 or 0.05) by comparing tumor samples with normal mammary gland controls in each subtype. We then carried out enrichment analysis on genes with hypo- or hyper-methylated promoter in the luminal B subtype, which is most closely recapitulated by the PyMT mice^26,27^. Genes with promoter hypermethylated DMRs were enriched in PRC2 targets (Fig. 5B). There were 313 PCR2 targets and their expression was significantly reduced when compared with all PRC2 targets (Supplementary Fig. S3A, Wilcoxon-test *p*-value < 0.05). The enrichment of PRC2 target genes was a common signature for other subtypes: we found 271, 206 and 43 PRC2 targets with promoter hypermethylation in luminal A, HER2-enriched and basal-like subtypes, with a significant overlap among these genes (Supplementary Fig. S3B).

To narrow down the candidate gene list, we used 12 PRC2 target genes overlapped between 60 PRC2 targets (DMR methylation difference ≥40% and FDR < 0.01) and 130 PRC2 targets with significantly reduced expression (FDR < 0.05) as a gene panel to test if they can predict metastasis risk in human patients. We applied the gene list to Gene Expression-Based Outcome for Breast Cancer (GOBO)^39^ and found there was a significant difference on distant metastasis-free survival (DMFS) between patients with high and low expression of those genes (Supplementary Fig. S3C). This suggests potential biological importance of DNA methylation mediated gene silencing in breast cancer. Because many PRC2 targets function in cell differentiation/differentiation, we further explored six such genes (*SLIT2, PDGFRA, GATA6, TAL1, FLI1, KLF4*) among the 12 genes; only *KLF4* is known to be involved in breast cancer based on text mining. They also presented prognostic value in breast cancer patients (Supplementary Fig. S3D), hence can be potential novel biomarkers that worth further investigation.

## Discussion

We observed, for the first time, a global dysregulation of DNA methylation during malignant breast cancer progression in the MMTV-PyMT mouse model. In addition to a global hypomethylation and possible preservation of DMCs, we observed an increase of both DMCs number and degree of methylation difference during tumor development. Such shift accompanied the malignant transition, with a slight overall shift towards hypermethylation. PRC2 target genes were the primary targets of this epigenetic field defects, these findings may contribute to explain the regulatory mechanisms underlying in breast cancer of PyMT mice and humans.

Based on the status of hormone receptors such as estrogen receptor (ER), progesterone receptor (PR), and human epidermal growth factor receptor-2 (HER2), breast cancers can be largely clustered into five subtypes: luminal A, luminal B (represented by the PyMT mouse model), HER2-enriched, basal-like, and normal-like^40^. The breast cancer hormone receptor status could be connection to DNA methylation^41,42^. Thus, in addition to these hormone receptors, DNA methylation also exhibit subtype-specific patterns, which has been shown in studies of cell lines, tissue samples, and TCGA data. For example, Park *et al*. reported distinct promoter CpG island methylation among breast cancer subtypes at 12 loci related to tumor progression, including *APC*, *DLEC1*, *GRIN2B*, *GSTP1*, *HOXA1*, *MT1G*, *RARB*, *RASSF1A*, *RUNX3*, *SCGB3A1*, *SFRP1*, and *TMEFF2*^43^. Dedeurwaerder *et al*. profiled DNA methylation in 248 breast tissues and characterized new breast cancer subtypes associated with T lymphocyte infiltration beyond the widely-used cancer classification based on gene expression profiles^44^. In the initial TCGA BRCA study, epigenetic changes in more than 800 breast tumors were detected^19^. Hierarchical clustering of methylation values of all samples revealed several groups corresponding to intrinsic subtypes, with one group showing hypermethylation significantly enriched for luminal B subtype samples and with low frequency of PIK3CA, MAP3K1 and MAP2K4 mutations. Some 490 genes, enriched in Wnt signaling, exhibited both DNA hypermethylation and reduced expression.

The higher methylation in luminal B samples has been also observed previously. In one study^45^, DNA methylation difference among luminal A, luminal B and basal-like subtype was readily observed in 189 breast tumor samples with basal-like ones being least frequently methylated. In particular, targets of PRC2 (Polycomb repressive complex) showed higher methylation in luminal B samples. In another^17^, 15 CpG loci were found differentially methylated among various subtypes and novel epigenotypes that distinguish basal-like tumors from HER2-overexpressing tumors. In a more recent study^46^, the unsupervised clustering of 188 breast tumor samples revealed seven DNA methylation epigenetic subgroups as epitypes. Only hypermethylation patterns in luminal subtypes might play roles in tumor progression but not that in basal-like subtype. In addition, studies of breast cancer cell lines have also identified distinct methylation patterns on CpG island shores among subtypes as well as hypomethylation in the basal-like B subtype^47,48^.

The study by Fang *et al*.^49^ has established foundations of epigenetic changes in the metastatic process of breast caner and showed that breast cancer can be characterized by B-CIMP (breast CpG isalnd methylator phenotype). The aforementioned study as well showed elevated methylation levels and frequencies in some gene promoters as well as a gradual increase in the number of methylated genes from normal mammary tissue to FEA, ADH and DCIS^23^. Here we report a global dysregulation of DNA methylation starting as early as the hyperplasia stage. Moreover, our previous analysis showed that genes such as *Dnmt1* implicated in DNA methylation were differentially expressed between PyMT samples and controls^30^. Although no significant DNA methylation changes was found in DNMTs and TETs genes, which are all key players in DNA methylation process, we observed an increased expression of DNMT3B in late carcinoma stage (log2 fold change is 1.28, FDR = 0.02) from a previous study of mRNA expression in this mouse model^30^. Together all provided strong evidence for interplay between dysregulation of DNA methylation and abnormal gene expression in tumorigenesis of breast cancer.

We also observed a similar enrichment of PRC2 targets among genes with promoter hypermethylation in the PyMT mice as well as in human breast cancer. This is very much in consistent with the findings in normal tissue adjacent to breast cancer exhibiting DNA methylation alterations, which are also associated with PRC2 targets^24^. Previously, cancer specific promoter DNA hypermethylation acossicated with Polycomb targets was observed^50–52^. In our study, we extended the observations to early stage lesions. It has been found that DNA methylation affects Polycomb target genes prior to cervical neoplastic transformation, and such risk can be predicted^53^. Recently, another study also found genes with promoter methylation alterations starting in early stage of cervical intraepithelial neoplasia were enriched in PRC2 targets^54^.

We hypothesized that there is a regulatory cascade from E2Fs to PRC2-mediated gene silencing, which connected our findings from both expression profiles and DNA methylation profiles. Two core components of PRC2 – EED and EZH2 – are targets of E2Fs^55,56^. Together with the E2Fs, they showed increased mRNA expression in PyMT samples. As discussed before, we also found elevated expression of *Dnmt1*, which is an E2F target as well^55,57^. The elevated E2Fs levels may result in increased expression of DNMT1, EED and EZH2, leading to reduced expression of PRC2 targets.

SUZ12, EED, and EZH2 are three core components of PRC2. EZH2 catalyzes histone H3 trimethylation of lysine 27 (H3K27me3), leading to transcriptional repression. EZH2 and SUZ12 are known to initiate tumorigenesis, and are overexpressed in various human cancers^58–60^. Increased expression of *EED* and *EZH2* was detected in human breast cancer lymph node metastases^61^. Overexpression of *EZH2* was as well reported in prostate cancer and is suggested to be involved in tumor progression^62^. It is also considered a marker of tumor aggressiveness in breast cancer^63^. Increased expression of PRC2 core subunits may promote malignant progression through EMT (epithelial–mesenchymal transition) by repressing E-cadherin^64–66^. The results of EZH2 high activation seem to be context specific: the molecule can transcriptionally silence DNA-damage repair genes, pRB tumor suppressor and lineage specification genes, all leading to cancer as a common consequence^67^. Many PRC2 targets are involved in differentiation, and the fact that EZH2 suppresses expression of some lineage specification genes suggests that EZH2 may promote transformation by repressing differentiation^67^. In our study, we also observed reduced expression of some PRC2 targets fuctioning in differentiation in PyMT mice.

It is known that DNA methylation and H3K27me3 are both involved in epigenetic gene silencing. In general, H3K27me3 and DNA methylation are mutually exclusive at CpG islands^68^, but the two repressive marks are more likely to co-occur at CpG islands and TSS of silenced genes in cancer^69^. Although the relationship between PRC2-mediated gene silencing and DNA methylation remains elusive, it seems to be more than a dual repression. In one study, epigenetic repression occurs first through gain of H3K27me3 and then through gain of DNA methylation^70^. In another study with mouse embryonic stem cells (ESCs), PRC2 is required for DNA methylation at some genes, while DNA methylation represses H3K27me3 placement globally^71^. Although EZH2 may recruit DNMTs^72^, it is suggested that the recruitment of DNMT3A to specific sites by EZH2 alone may not be sufficient for *de novo* DNA methylation^73^. Multiple factors, such as transcription factors and histone modification are involved in recruiting PRC2 to targeted genomic regions^74^. Studies also suggested that the long non-coding RNA (lncRNA) *HOTAIR* interacts with PRC2 and is required to target its occupancy^75^. In breast cancer, *HOTAIR* showed increased expression in primary tumors as well as metastases^76^. In addition to lncRNA, another type of non-coding RNAs: microRNA, also contribute to breast cancer progression in this PyMT mice as sugguested by our previous study^77^. Taken together, there seems be a synergy of epigentic regulation and gene expression in the PyMT mouse model of breast cancer.

Although our current findings need further validation and more studies to understand the functional links between E2Fs-(PRC2/DNMTs)-PRC2 targets repression, it is important to point out that EZH2 and DNMT1 are targeted by multiple drugs (EZH2: EI1, EPZ-6438, GSK126; DNMT1: PROCAINAMIDE, IFOSFAMIDE, DECITABINE, CISPLATINUM) according to DGIdb (Drug-Gene interaction database)^78^. The elevated expression of both genes may warrant a combinatory use for breast cancer early treatment and metastasis prevention. The findings from this study help to improve our understanding of the molecular mechanisms of breast cancer progression in the MMTV-PyMT mouse model as well as in humans. Combining it with our previous mRNA study, we found that gene expression and DNA methylation changes precede tumor malignant transition in this mouse model. New breast cancer candidate genes discovered in both studies warrant further investigation and may serve as cancer biomarkers for prognosis.

## Methods

### Animals and tissue collection

This study was approved by the Institutional Animal Care and Use Committee (IACUC) of Albert Einstein College of Medicine. All procedures involving mice were conducted in accordance with the National Institutes of Health guidelines concerning the use and care of experimental animals. Male PyMT mice (FVB/N-Tg (MMTV-PyVT) 634Mul/J mice, Stock Number: 002374, the Jackson Laboratory) were randomly bred with homozygous FVB females to obtain F1 female mice (PyMT mice hereafter). They were heterozygous for the PyMT transgene, they developed breast cancer spontaneously and were used as cases. Age matched homozygous FVB females were used as controls.

We selected four time points, corresponding to the main stages of tumor progression: most PyMT mice develop hyperplasia at week 6, adenoma/MIN at week 8, early carcinoma at week 10, and late carcinoma with lung metastasis at week 12^25^. At each time point, three PyMT mice and three age-matched controls were sacrificed, bulk mammary tumors and normal mammary glands were collected, snap froze and stored at −80 °C until use. The histological changes undergoing in the PyMT samples dissected for DNA methylation analysis was evaluated by a pathologist after H&E staining. Typical features of the tumor developmental stage based on the mouse age were observed (see our previous paper^30^ for details. Samples used for RNA-sequencing and ERRBS were not necessarily from the same tumor piece.)

### Enhanced Reduced Representation Bisulphite Sequencing (ERRBS)

Total genomic DNA was extracted from the snap frozen samples using a phenol-chloroform method^79^. DNA samples passing Nanodrop quality control were used for ERRBS following the standard protocol^3,80^.

### Data preprocessing and detection of differential methylation

Raw reads passing Illumina’s purity filter were preprocessed by an ERRBS pipeline^80^, which uses Bismark^81^ to align reads to the mm10 reference genome. All statistical analysis were carried out by R v3.2.1^82^. After assessing samples quality with reads coverage, methylation percentage, and principal component analysis, we removed two outliers from the dataset.

Using the methylKit R package^83^ we analyzed DMCs. We first retained in the analysis reads with ≥10 coverage and captured, on average, ~1.4 million confident loci. For each locus, we then calculated a methylation score as percentage of methylation (number of methylated cytosines/(number of methylated cytosines + unmethylated cytosines)) and got the methylation difference between PyMT samples and age-matched FVB control samples. We mapped DMCs (methylation difference ≥10% and *q*-value < 0.01) to genes, using annotation from the UCSC genome browser (https://genome.ucsc.edu). Promoters are defined as RefSeq transcription start site (TSS) ±2 kb. Biological pathways and gene sets enrichment were performed using GSEA^31^. Predicted mouse enhancer regions were obtained from a study using the mouse ENCODE data^84^; enhancer prediction was based on ChIP-seq data of multiple tissues and cell types^84^. Data originally mapped to mm9 was lifted over to mm10 using the R package rtracklayer. Permutation tests were used to demonstrate the significance of DMCs enrichment. For permutation tests, we randomly sampled *n* loci of interest (for example, number of DMCs), and count how many mapped to annotation *X* (for example, enhancers); the process was repeated 1,000 times to generate an empirical distribution of overlap counts and determine a *p*-value.

### Co-methylation network analysis

We adopted methods from the WGCNA (weighted gene co-expression network analysis)^85^ to study gene co-methylation network. We first retained significant DMCs (*q*-value < 0.01) from previous step. Next, for computational efficiency, we further restricted to DMCs in promoter regions 2 kb upstream to TSS. We focused on upstream promoter because the GETx eQTL study^86^ showed an upstream bias (more eQTLs located upstream of TSS), suggesting upstream TSS regions possibly have greater influence on expression. After these two steps, over 21k CpG loci remained and were used to construct a signed co-methylaiton network using an adjacency matrix based on Pearson’s correlation coefficient on every pair of CpG loci. Modules were identified using a “dynamic tree cut” method^87^. Preservation of module connectivity patterns between cases and controls were gauged by a z-summary statistic. Modules with high preservation showed significant difference on module eigengene (first principal component of a module) between PyMT samples and controls (Wilcoxon-test adjusted *p*-value < 0.05), meaning methylation values varied in the two groups.

### Bisulphite MassArray verification assays

We performed Bisulfite MassArray to verify our ERRBS results using samples from carcinoma stage. We bisulfite-converted DNA using the Zymo EZ-Methylation-Gold Kit. Primers were designed to span loci with high, intermediate and low levels of methylation (Supplementary Table S3). We performed MassArray (EpiTyper) and proved a high correlation between MassArray and ERRBS results (Supplementary Fig. S4).

### Availability of Data

The PyMT ERRBS data is available in the Gene Expression Omnibus (GEO) database as GSE83623.

### Declarations and Ethics Approval

This study of cancer in mice was approved by the Institutional Animal Care and Use Committee (IACUC) of Albert Einstein College of Medicine. All procedures involving mice were conducted in accordance with the National Institutes of Health guidelines concerning the use and care of experimental animals.

## Electronic supplementary material

Supplementary Table and Figures

Dataset 1
