# Supplementary material for: Epigenetic alterations to Polycomb targets precede malignant transition in a mouse model of breast cancer
Source: Sci Rep. 2018 Apr 3;8:5535. doi: 10.1038/s41598-018-24005-x (PMC5882905; doi:10.1038/s41598-018-24005-x)
Supplement: Supplementary file 1 — Supplementary Table and Figures [file 41598_2018_24005_MOESM1_ESM.docx]

**Epigenetic alterations to Polycomb targets precede malignant transition in a mouse model of breast cancer**

Ying Cai^1^ (ying.cai@phd.einstein.yu.edu), Jhih-Rong Lin^1^ (jhih-rong.lin@einstein.yu.edu), Quanwei Zhang^1^ (quanwei.zhang@einstein.yu.edu), Kelly O’Brien^1^ (kobrien822@gmail.com), Cristina Montagna^1,2^ (cristina.montagna@einstein.yu.edu), Zhengdong D. Zhang^1,*^ (zhengdong.zhang@einstein.yu.edu)

# Supplementary FILES

Supplementary Table S1 (in Excel file)

Supplementary Figures S1-4

#

Supplementary Table S1. Primer design for MassArray verification. (Excel)

Supplementary Figures


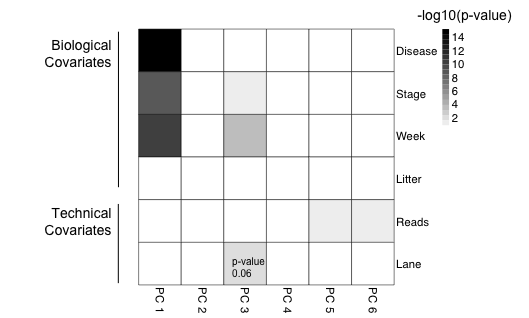


Figure S1. Analysis of sources of variability. Color intensity represents contribution significance. The heatmap shows –log_10_ *p*-value of ANOVA linear modeling on top PCs accounting for some known covariates.

Figure S2. Components of CpG loci and permutation tests. (A) Composition of all CpG loci captured and DMCs mapped to enhancers. (B) The permutation test of the enrichment of DMCs in enhancers. The grey peak is the null distribution generated by random permutation. The red line is the observed enrichment of DMCs in enhancers. (C) Composition of DMCs and hypermethylated DMCs mapped to promoters. (D) The permutation test of the enrichment of hypermethylated DMCs in promoters. H= hyperplasia, A= adenoma/MIN, EC= early carcinoma, LC= late carcinoma.


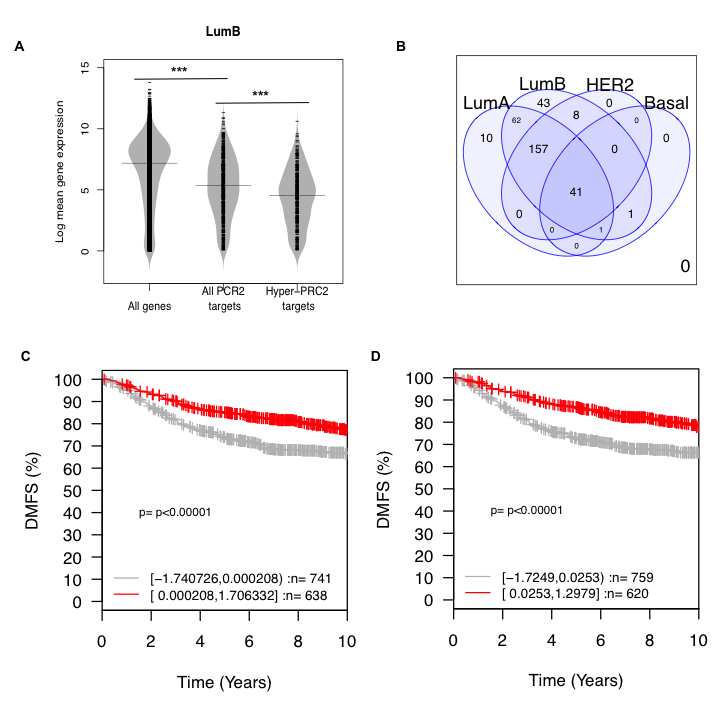


Figure S3. TCGA data validation for PRC2 target genes. (A) Violin plot of log transformed mean gene expression in luminal B samples. There was significant difference between expression of all PRC2 targets and those PRC2 targets affected by promoter hypermethylation (***: Wilcoxon-test *p*-value < 0.05). (B) Venn plot for overlapping PRC2 targets among different breast cancer subtypes. Numbers are PRC2 target genes with promoter hypermethylation found in each subtype. (C) Kaplan-Meier survival analysis using the 12-gene panel in breast cancer patients. (D) Kaplan-Meier survival analysis using the 6-gene panel in breast cancer patients.


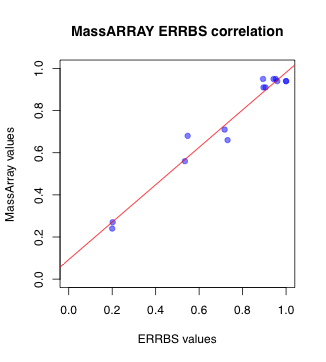


Figure S4. Verification of ERRBS by MassArray. There is a high correlation between MassArray values and the values from ERRBS.

# 
